# Myeloid-Derived Suppressor Cells and Clinical Outcomes in Children With COVID-19

**DOI:** 10.3389/fped.2022.893045

**Published:** 2022-06-06

**Authors:** Katherine Bline, Angel Andrews, Melissa Moore-Clingenpeel, Sara Mertz, Fang Ye, Victoria Best, Rouba Sayegh, Cristina Tomatis-Souverbielle, Ana M. Quintero, Zachary Maynard, Rebecca Glowinski, Asuncion Mejias, Octavio Ramilo

**Affiliations:** ^1^Center for Vaccines and Immunity, Nationwide Children’s Hospital, Columbus, OH, United States; ^2^Division of Critical Care Medicine, Nationwide Children’s Hospital, Columbus, OH, United States; ^3^Abigail Wexner Research Institute, Nationwide Children’s Hospital, Columbus, OH, United States; ^4^Division of Infectious Disease, Nationwide Children’s Hospital, Columbus, OH, United States

**Keywords:** myeloid-derived suppressor cell (MDSC), pediatric, COVID-19, respiratory disease, T cell, immune function

## Abstract

**Background:**

Although children with COVID-19 account for fewer hospitalizations than adults, many develop severe disease requiring intensive care treatment. Critical illness due to COVID-19 has been associated with lymphopenia and functional immune suppression. Myeloid-derived suppressor cells (MDSCs) potently suppress T cells and are significantly increased in adults with severe COVID-19. The role of MDSCs in the immune response of children with COVID-19 is unknown.

**Aims:**

We hypothesized that children with severe COVID-19 will have expansion of MDSC populations compared to those with milder disease, and that higher proportions of MDSCs will correlate with clinical outcomes.

**Methods:**

We conducted a prospective, observational study on a convenience sample of children hospitalized with PCR-confirmed COVID-19 and pre-pandemic, uninfected healthy controls (HC). Blood samples were obtained within 48 h of admission and analyzed for MDSCs, T cells, and natural killer (NK) cells by flow cytometry. Demographic information and clinical outcomes were obtained from the electronic medical record and a dedicated survey built for this study.

**Results:**

Fifty children admitted to the hospital were enrolled; 28 diagnosed with symptomatic COVID-19 (10 requiring ICU admission) and 22 detected by universal screening (6 requiring ICU admission). We found that children with severe COVID-19 had a significantly higher percentage of MDSCs than those admitted to the ward and uninfected healthy controls. Increased percentages of MDSCs in peripheral blood mononuclear cells (PBMC) were associated with CD4+ T cell lymphopenia. MDSC expansion was associated with longer hospitalizations and need for respiratory support in children admitted with acute COVID-19.

**Conclusion:**

These findings suggest that MDSCs are part of the dysregulated immune responses observed in children with severe COVID-19 and may play a role in disease pathogenesis. Future mechanistic studies are required to further understand the function of MDSCs in the setting of SARS-CoV-2 infection in children.

## Introduction

At the end of 2019, SARS-CoV-2 originated a pandemic that is anticipated to remain endemic. Although adults account for the vast majority of hospitalizations and fatalities attributed to SARS-CoV-2, children also develop severe disease requiring hospitalization. Of those children admitted to the hospital, almost one-third require admission to the intensive care unit (ICU) (1). In adults, severe coronavirus disease of 2019 (COVID-19) induces a dysregulated host immune response that is characterized by concurrent hyperinflammatory and anti-inflammatory responses. The inflammatory cytokine profiles in adults with COVID-19 requiring ICU admission are similar to those elicited in patients with acute respiratory distress syndrome or sepsis (2). Additionally, T cell lymphopenia and functional impairment are also common among critically ill adults infected with SARS-CoV-2 and are associated with more severe illness and increased risk of death (3, 4). The underlying immunological mechanisms that contribute to the heterogenous phenotypes of SARS-CoV-2 infection in children are ill-defined.

Myeloid-derived suppressor cells (MDSCs) are immature, heterogenous cells that appear to be a major determinant of the dysregulated host response to SARS-CoV-2 in adults. MDSCs are expanded during inflammatory conditions and potently suppress T cell proliferation and cytokine production contributing to adaptive immune suppression (5, 6). In adult patients with malignant tumors, inhibition of MDSCs is associated with improved T cell function, decreased tumor burden, and improved clinical outcomes (7, 8). The two predominant subsets of MDSCs, granulocytic (G-MDSCs) and monocytic (M-MDSCs), each suppress T cells by a variety of mechanisms, including upregulation of cell surface marker programmed death-ligand 1 (PD-L1) (9, 10). MDSCs also mediate immune suppression through induction of T regulatory cells and inhibition of natural killers (NK) cells (11, 12). In adults with severe COVID-19, MDSC expansion has been associated with increased risk of death (13–15). It is unknown if MDSCs are also expanded in children with acute SARS-CoV-2 infection and if they play a role in disease pathogenesis. We hypothesize that children with severe COVID-19 will have an expansion of MDSC populations that will be associated with clinical outcomes.

## Materials and Methods

### Study Population and Design

This is a prospective, observational study conducted at Nationwide Children’s Hospital (NCH) in Columbus, OH, a free-standing children’s hospital. We enrolled a convenience sample of children with COVID-19. Subjects of all ages were eligible if they were admitted to the ward or pediatric intensive care unit (PICU) at NCH from November 2020 to December 2021 and diagnosed with SARS-CoV-2 infection confirmed by polymerase chain reaction (PCR). Subjects were approached if their native language included one of the following: Arabic, Chinese, Croatian, French, German, Spanish, Nepali, Polish, Russian, Somali, and Kinyarwanda. A hospital-employed or approved interpreter was used to review the full consent process and the families were also provided a short form to read in their native language (Supplementary Figure 3). Exclusion criteria included children with a limitation of care order in place at the time of enrollment, a diagnosis of malignancy or primary immunosuppression, or those with multi-system inflammatory syndrome in children (MIS-C) as defined by the Center for Disease Control (16), as MIS-C reflects a post-acute sequelae of SARS-CoV-2 infection. Initial disease severity for subjects admitted to the PICU was measured using Pediatric Risk of Mortality (PRISM) III (17) and Pediatric Logistic Organ Dysfunction (PELOD)-2 scores (18).

A cohort of pre-pandemic uninfected healthy controls (HC) presenting to the hospital for imaging studies were also included for comparative purposes. HC samples were collected from July 2018 to December 2019, subjects were excluded if febrile within 24 h prior to enrollment, receiving antibiotic treatment, had an oncologic diagnosis, were a transplant recipient, or were prescribed immunomodulatory medications. Data from the healthy control cohort has not been previously published. Informed consent was obtained from the subjects’ legal guardians and, when appropriate, assent was obtained from the subject. The protocol was approved by the Institutional Review Board at Nationwide Children’s Hospital (IRB STUDY00000921).

### Data and Sample Collection

#### Data and Blood Samples

Demographic and clinical data for study subjects were collected using a dedicated database built for the study and from the electronic healthcare records. All data were recorded and stored in password protected electronic case report forms as part of Research Electronic Data Capture (REDCap). Data collected included demographic information and comorbidities, complete blood count (CBC), respiratory support, COVID-19-directed therapies received, and duration of hospitalization. Protected health information was kept separate from clinical data. The initial blood sample for hospitalized subjects was obtained at time of consent within 48 h of hospital admission to the ward or ICU. SARS-CoV-2 infection was diagnosed per standard of care using a PCR assay in nasopharyngeal (NP) samples including: GeneXpert Xpress SARS-CoV-2; Cepheid, Sunnyvale, California, the FilmArray Respiratory Viral Panel (BioFire, Salt Lake City, Utah), or SARS-CoV-2 PCR using US centers for Disease Control and Prevention 2019-nCoV_N1 primers and probes (19). Blood sampling from HC was performed at the time of intravenous catheter placement for imaging studies.

#### Peripheral Blood Mononuclear Cells Staining

Blood was collected in acid citrate dextrose (ACD) tubes and, within 2 h of sampling, peripheral blood mononuclear cells (PBMC) were isolated from whole blood by density gradient centrifugation at 530 g for 30 min at room temperature (Ficoll-Hypaque; Sigma-Aldrich, St. Louis, MO). If sufficient quantity available, one million cells were reserved for myeloid-derived suppressor cell (MDSC) staining for flow cytometric analysis. Cells were stained for 20 min at 4°C for the following cell surface markers: CD45 conjugated to BUV 395 (BD Biosciences, San Jose, CA), HLA-DR conjugated to BV 711, CD3 conjugated to FITC, CD4 conjugated to PerCP-Cy55, CD127 conjugated to BV 421, CD14 conjugated to BV 510, CD38 conjugated to BV605, CD11b conjugated to BV785, PD-L1 conjugated to PE, CD56 conjugated to PE Cy5, CD33 conjugated to PE Cy7, CD25 conjugated to APC, CD15 conjugated to AF700, and CD8 conjugated to APC Fire 750 (all except CD45 from BioLegend, San Diego, CA). Cells were washed and then fixed using 4.21% paraformaldehyde fixation buffer for 30 min at 4°C (BD Biosciences, San Diego, CA). Samples were stored at 4°C and acquired using a BD LSRFortessa Flow Cytometer (BD Biosciences) within 4 days of staining. Compensation control beads were used to correct for fluorescence spectral overlap. Flourescence Minus One (FMO) controls were used to optimize gating strategy. Data analysis was performed using FlowJo 10.7.1 (BD Biosciences).

MDSCs were identified using the following gating strategy: CD45 positive, HLA-DR negative, and both CD33 and CD11b positive; MDSC subsets were further identified as M-MDSCs (CD14 positive, CD15 negative) or G-MDSCs (CD15 positive, CD14 negative) (Supplementary Figure 1). MDSCs were quantified as a percentage of total PBMC; G-MDSC and M-MDSC were quantified as a percentage of total MDSCs. A cutoff of greater than 3% MDSC was used to define the upper limit of normal MDSC proportion based on previously published data demonstrating that healthy donors have less than 2% MDSC of circulating PBMC (20). T cells were identified as CD45+ and CD3+, CD3 + T cells were then used as the parent gate to identify CD4+ and CD8 + T cells. T regulatory cells were defined as CD3+/CD4+/CD25+ and CD127 low (Supplementary Figure 2). CD4+, CD8+, and T regulatory (Tregs) T cells were quantified as percentage of total CD3 + T cells. Activated T cells were defined as positive for CD38 and HLA-DR and quantified as percentage of respective CD4+, CD8+ or T regulatory cells. NK cells were identified as CD3−/CD56+ (Supplementary Figure 2) and quantified as a percentage of total PBMC.

### Statistical Analysis

Data are summarized using frequencies and percentages for categorical variables and medians with 25–75% interquartile range for continuous variables. Differences between groups were evaluated using chi-square or Fisher’s exact tests for categorical variables and rank-sum tests for continuous variables. For all tests, a two-tailed *p*-value < 0.05 was considered significant. Associations with MDSCs were evaluated using Spearman correlation coefficients. Values from ± 0.1 to 0.3 indicate a marginal association, from ± 0.3 to 0.5 represent a moderate association, and > 0.5 indicate a strong association. Effect sizes are summarized using standardized differences. Because the sample size within groups is small and there are many comparisons, *p*-values may not detect true differences among groups. Instead, it is recommended to focus on standardized differences (21). In addition, comparisons by high MDSCs (using the threshold of > 3%) were evaluated using chi-square or Fisher’s exact tests for categorical variables and rank-sum tests for continuous variables. We used GraphPad Prism Version 7.0 for Windows (GraphPad Software, La Jolla, California, United States) for statistical analyses.

## Results

### Study Subjects

**FIGURE 1:**
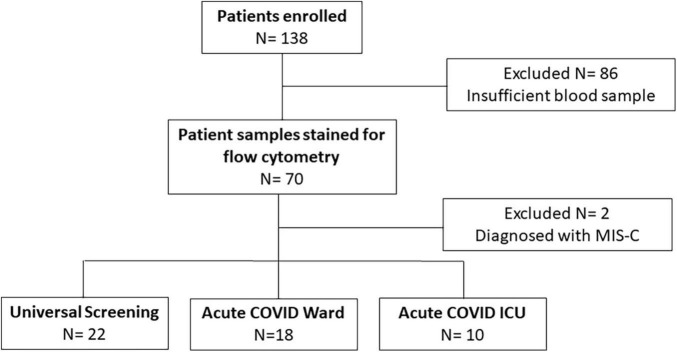
Enrollment flow diagram. This flow chart diagrams enrollment, reasons for exclusion, and final numbers for three comparison groups.

We enrolled a convenience sample of 138 subjects (ages 3 weeks to 21 years) hospitalized between November 2020 and December 2021 with positive SARS-CoV-2 PCR, of which 86 were excluded for insufficient blood sample and 2 with an MIS-C diagnosis (Figure 1). Of the remaining 50 subjects enrolled, 28 children were diagnosed with symptomatic COVID-19 (18 were admitted to the inpatient ward and 10 children had severe illness defined by the need for intensive care level treatment) and 22 were identified *via* universal screening. Two subjects in the symptomatic COVID-19 group had a diagnosis of adrenal insufficiency requiring chronic steroid treatment. In the screening cohort, 6 subjects were admitted to the PICU: severe trauma (*N* = 2, including gunshot wound and high speed motor vehicle crash), infective endocarditis (*N* = 1), acute diabetic ketoacidosis (*N* = 1), post-operative monitoring (*N* = 1), and intentional ingestion (*N* = 1). Those admitted to the ward found to be incidentally SARS-CoV-2 positive included children with abscesses (*N* = 2), appendicitis (*N* = 3), MVC (*N* = 1), pelvic inflammatory disease (*N* = 1), eczema herpeticum (*N* = 1), bone fracture (*N* = 1), pituitary adenoma (*N* = 1), failure to thrive (*N* = 1), intentional ingestion (*N* = 1), inflammatory bowel disease (*N* = 1), chronic parotitis (*N* = 1), moyamoya complications (*N* = 1), and functional neurologic disorder (*N* = 1). Children admitted to the PICU with either acute COVID-19 or incidentally positive as part of the screening cohort had similarly low severities of illness indicated by PRISM III and PELOD-2 scores (1.5 [0, 5] vs. 3 [0, 10], *p* = 0.5, and 3 [2, 7.5] vs. 5 [2, 8], *p* > 0.99, respectively). Children in the screening cohort (ICU and ward) were older (median age 13 [9, 16] years) and had longer hospitalizations (median 99 [50, 186] days) compared with children with acute COVID-19 (Table 1). There were no significant differences in sex, race/ethnicity, or SARS-CoV-2 vaccination status among the three groups (Table 1).

**TABLE 1:** Characteristics of children with SARS-CoV-2 and healthy controls.

Characteristics	COVID ICU (*N* = 10)		COVID ward (*N* = 18)		Universal screening (*N* = 22)		Healthy controls (*N* = 30)		
		
	Median or *N*	IQR or %	Median or *N*	IQR or %	Median or *N*	IQR or %	Median or *N*	IQR or %	*p*-value
Age, years	8.8	[1.0, 22.3]	3.2	[0.7, 11.9]	13	[9,16]	6.1	[4.1, 8.1]	0.0084

Female	4	40%	7	39%	13	59%	11	36.7	0.62

Race									
White	8	80%	10	55%	13	59%	24	80%	
Black	2	20%	4	22%	4	18%	2	7%	>0.99
Multiracial			2	11%	2	9%	1	3%	
Other			1	6%	3	14%	1	3%	

Ethnicity									>0.99
Hispanic or latino	0	0%	1	5%	4	18%	2	7%	
Not hispanic or latino	10	100%	17	95%	18	82%	28	93%	

Underlying conditions									
Obesity	3	30%	4	22%	8	36%	1	3%	
Respiratory	2	20%	4	22%	1	5%	6	20%	
Cardiac	2	20%	1	6%	2	6%	5	17%	>0.5
Genetic/Neurologic	4	40%	3	17%	2	6%	10	33%	
Other*^a^*	0	0%	5	28%	2	6%			

Days of Symptoms	2.5	[2, 5.8]	4	[2.3, 7]	NA		NA		0.35

Vaccination Status							NA		
Vaccinated	2	20%	2	11%	5	23%			
Unvaccinated	5	50%	10	55%	3	14%			0.63
Not known	3	30%	6	33%	14	64%			

Initial PRISM III Score	1.5	[0, 5]	NA		3^#^	[0,10]	NA		0.5

PELOD-2 Score*	3	[2, 7.5]	NA		5^#^	[2,8]	NA		>0.99

COVID-directed therapy									
Steroid	80^∧^	80%	3^∧∧^	17%	NA		NA		0.0005
IVIG	0	0	1	6%					>0.99
Remdesivir	5	50%	0	0					0.0026

Hospitalization, days	9.9	[5.4, 22.2]	1.4	[0.9, 2.1]	99	[50, 186]	NA		<0.0001
									

PRISM III, Pediatric Risk of Mortality score; PELOD, Pediatric Logistic Organ Dysfunction score, IVIG, intravenous immune globulin; *highest value within first 48 hours of admission. ^#^Represent the PRISM III and PELOD 2 scores of the 6 universal screening subjects admitted to the ICU. ^∧^Indicates 6 subjects received prior to blood sample; ^∧∧^indicates 3 subjects received prior to blood sample. Values in bold indicate significant P-values.

*^a^Other includes renal disease (n = 2), inflammatory bowel disease (n = 1), diabetes (n = 1), and sickle cell disease (n = 1) in the COVID-19 cohort, and renal disease (n = 1) and autoimmune disease (n = 1) in the universal screening cohort.*

Children with acute COVID admitted to the ICU had similar duration of symptoms as those admitted to the floor but were more likely to receive steroids or remdesivir as part of COVID-directed treatment (Table 1). In all children with acute COVID-19 (including subjects admitted to ICU and ward), obesity and genetic/neurologic diseases were the most prevalent underlying chronic conditions, (*N* = 7 of 28, for each disease). However, there was not a significant difference in underlying conditions when comparing subjects with symptomatic COVID-19 admitted to the ICU vs. the ward. Additionally, asthma and chronic lung disease were common in children with symptomatic COVID-19, including 20% of those admitted to the ICU and 22% of those admitted to the ward. Of the children with acute COVID-19 admitted to the ICU, 6 (60%) received steroids before the initial sample compared with 3 (17%) subjects admitted to the ward. Duration of hospitalization was longer in children admitted to the ICU compared with that of children hospitalized on the ward. In the universal screening and healthy control cohorts, 36 and 3% of children were obese, respectively.

### Differences in Myeloid-Derived Suppressor Cells According to Study Groups

**FIGURE 2:**
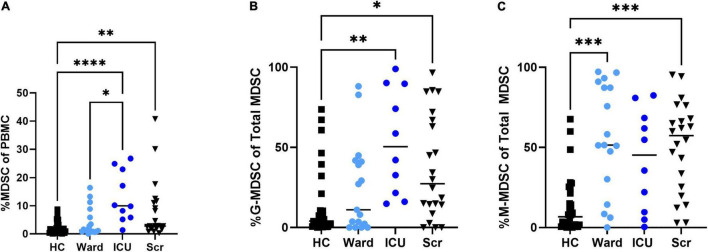
Total MDSC and G- and M-MDSC subsets in children with acute COVID-19 compared to healthy controls and universal screening (Scr) subjects. Children critically ill due to acute COVID-19 had increased proportion of (A) Total (T-) MDSCs and (B) granulocytic (G-) MDSCs compared to those with less severe illness and healthy controls; T-MDSC were also increased in the Scr cohort compared to HC. (C)There was no significant difference in the proportion of monocytic (M)-MDSC between those children admitted to the floor or ICU with symptomatic COVID-19, but children admitted to the ward and in the Scr cohort had an increased proportion of M-MDSC compared to healthy controls. Kruskal-Wallis test was performed, data represent median with IQR. HC (*N* = 30), Ward (*N* = 18), ICU (*N* = 10), Scr (*N* = 22). *p-value < 0.05, ^**^p-value < 0.01, ^***^p-value < 0.001, ^****^p-value < 0.0001.

**FIGURE 3:**
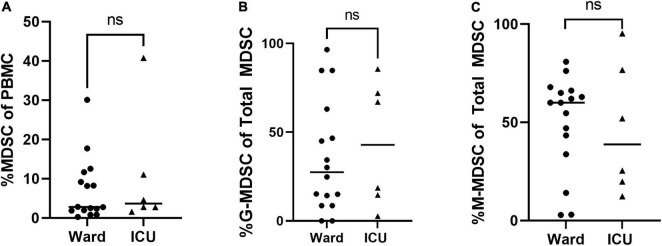
Total MDSC and G- and M-MDSC subsets in children positive on universal screening. In children SARS-CoV-2 positive but asymptomatic for symptoms of acute COVID, there were no significant differences in subjects admitted to the floor vs. ICU in (A) Total (T-) MDSCs and (B) granulocytic (G-) MDSCs or (C) monocytic (M-) MDSCs. Mann-Whitney test was performed, data represent median with IQR. Ward (*N* = 22), ICU (*N* = 6). NS indicates *p*-value > 0.05.

Overall percentages of MDSCs were significantly higher in children with COVID-19 compared to healthy controls. Furthermore, children with COVID-19 requiring admission to the PICU had a significantly increased proportion of MDSCs compared to children admitted to the ward (10.2% [8.23, 23.0] vs. 1.21% [0.8, 3.5], *p* = 0.02; Figure 2A), with granulocytic (G)-MDSCs being the predominant subtype (58.7% [22, 90] vs. 8% [0, 39], *p* = 0.005; Figure 2B). Monocytic (M)-MDSCs were increased in children admitted to the ward with acute COVID-19 and in those identified by universal screening compared to uninfected controls (Figure 2C). In the screening cohort, there were no significant differences in% total (T)- MDSC or subsets between children admitted to the ICU compared to the ward (Figure 3). In all children with acute COVID-19 (ward and ICU), expression of the cell surface marker programmed death-ligand 1 (PD-L1) was almost exclusively identified on G-MDSC compared to monocytic (M)-MDSC (100% [74, 100] vs. 1.4% [0.6, 6.3], *p* < 0.0001).

### Myeloid-Derived Suppressor Cells, T Cells, and Natural Killers Cells in Children With Symptomatic COVID

**FIGURE 4:**
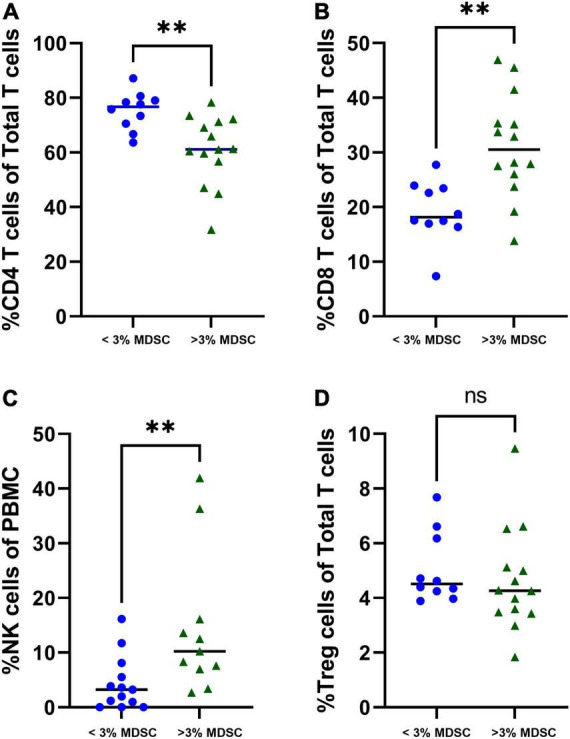
Associations with T-MDSC and immune cells in patients with symptomatic COVID-19. Increased frequencies of T-MDSC were associated with (A) decreased percentage of CD4 + T cells, and increased percentages of (B) CD8 + T cells and (C) NK cells. (D) There were no significant associations between T-MDSC frequency and percentage of T regulatory cells. < 3% MDSC (*N* = 10), > 3% MDSC (*N* = 14). Mann-Whitney test was performed, data represent medians with IQR values. ^**^p-value < 0.01.

Children with increased percentage of T-MDSC, defined as > 3% total MDSC of PBMC (20), were associated with a decreased percentage of CD4 + T cells (*p* = 0.002) (Figure 4A), and increased percentage of CD8 + T cells and NK cells (both *p* < 0.001) compared with to children with < 3% MDSCs (Figures 4B,C). There was not a significant difference in the percentage of T regulatory cells in children stratified by low or high MDSC (Figure 4D).

In children with severe COVID-19, there were stronger associations between the percentage of G-MDSC and other immune cells compared to those admitted to the ward. Notably, in children admitted to the ICU the percentage of G-MDSC was negatively associated the percentage of CD4 + T cells and positively associated with the percentage of CD8 + T cells and NK cells (Table 2). Both CD4 + and CD8 + T cells demonstrated increased expression of cell surface markers of activation (CD38 + and HLA-DR+) associated with G-MDSCs in children with acute COVID-19 requiring admission to the ICU (*r* = + 0.63, *r* = + 0.67, respectively). These associations between percentage of G-MDSC and particular immune cells were not observed in children admitted to the ward with symptomatic COVID-19. Of note, samples from four children admitted to the ward with acute COVID-19 were not available for T cell analysis due to processing error.

**TABLE 2:** Correlations with% G-MDSC in children with acute COVID.

	All		ICU		Ward	
Immune cell	rho	*p*-value	rho	*p*-value	rho	*p*-value
%CD4 T cells	−0.48	0.023	−**0.55**	0.125	−0.06	0.835
% CD4 T cells CD38 + HLA-DR +	0.39	0.073	**0.63**	0.067	0.06	0.842
%CD8 T cells	**0.60**	0.003	**0.72**	0.030	0.42	0.149
%CD8 T cells CD38 + HLADR +	0.41	0.059	**0.67**	0.050	−0.24	0.425
%T regulatory cells	−0.14	0.543	−0.35	0.356	0.17	0.573
CD4:CD8 ratio	−**0.57**	0.005	−**0.58**	0.099	−0.38	0.198
% NK cells	0.23	0.260	**0.60**	0.088	−0.15	0.588

*CD, cluster domain; HLA-DR, human leukocyte antigen-DR isotype; NK, Natural Killer. Values in bold indicate moderate or large effect size.*

### Associations With Myeloid-Derived Suppressor Cells and Clinical Outcomes

**FIGURE 5:**
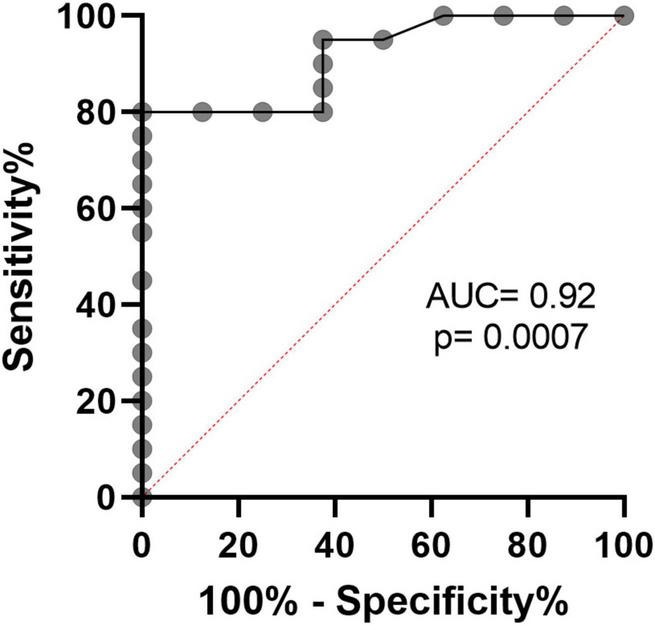
Receiver operating characteristics curve demonstrates that% T-MDSC is associated with need for respiratory support in children with symptomatic COVID. In all children with COVID, admitted to both ICU and the ward, increased % T-MDSC was associated with higher likelihood of requiring respiratory support (*N* = 8/28), AUC = 0.92, *p* = 0.0007.

**FIGURE 6:**
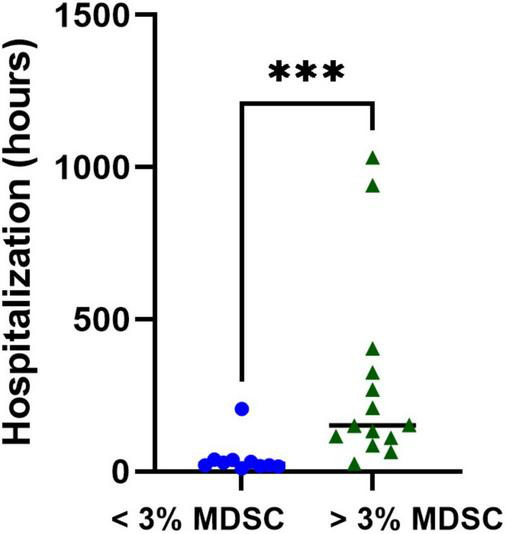
Associations with T-MDSC and duration of hospitalization in children with symptomatic SARS-CoV-2. Increased initial% T-MDSC was associated with longer hospitalization in children admitted with COVID compared to those with lower circulating % T-MDSC. Mann-Whitney test was performed, data represent medians with IQR values. ***p = 0.003.

Next, we calculated the receiver operating curve (ROC) characteristics to determine the association between% T-MDSC and the need for respiratory support. As shown in Figure 5, increased T-MDSC in children with acute COVID-19, admitted to both the ward and ICU, was significantly associated with a higher likelihood of requiring respiratory support (AUC 0.92, *p* = 0.0007). Of the 8 children requiring respiratory support, 4 children required intubation, 3 required non-invasive biphasic PPV (BiPAP), and 1 received supplemental oxygen *via* nasal cannula. Additionally, children with acute COVID-19, and > 3% T-MDSC had a longer duration of hospitalization compared to children with circulating MDSC closer to normal frequencies (Figure 6).

## Discussion

Since the beginning of the pandemic, over 100,000 children have been hospitalized and more than 1,000 have died in the United States alone due to SARS-CoV-2 infection (22, 23). The immune factors that are associated with severe COVID-19 in children are not well understood. Severe COVID-19 disease in adults has been characterized by lymphopenia and suppressed immune function (24). MDSC expansion is known to cause T cell lymphopenia and suppression and could be an important contributor to clinical outcomes in children with COVID-19. In this study, we found that children with COVID-19 have significantly increased percentages of MDSC that were associated with CD4 + T cell lymphopenia and worse clinical outcomes defined by increased need for respiratory support and prolonged hospitalization.

Initial investigations into the pathogenesis of SARS-CoV-2 infection in adults described a hyperinflammatory host response with associated cytokine storm as a key driver for disease severity and mortality (25). However, subsequent studies demonstrated that patients with severe COVID-19 have profound lymphopenia and decreased functional capacity of immune cells that was associated with increased risk of nosocomial infection and mortality (24, 26). MDSCs are increased as a result of host inflammatory conditions and suppress T cells and NK cells (27), and MDSCs have been found to be greatly increased in adults with severe COVID and associated with increased risk of mortality (15, 28, 29). Similar to studies in adults, we found that MDSCs were significantly increased in circulating peripheral blood of children, particularly G-MDSCs, and the expansion of these cells was associated with increased need for respiratory support, suggesting that they may play a role in COVID-19 lung disease.

A unique feature of our study was the inclusion of two other groups for comparison: uninfected healthy controls and children with COVID-19 identified by universal screening. Consistent with previous studies in adults, we found that children with severe COVID-19 had significantly increased proportions of circulating MDSCs compared to those admitted to the ward and the uninfected healthy controls. There were no significant differences in percentages of T-MDSC and G-MDSC between children in the ICU with COVID-19 and those identified by universal screening, likely because the screening group included children admitted to the ICU for various disease conditions that can be associated with significant increase in MDSCs [including severe trauma and diabetic ketoacidosis (30, 31)]. Additionally, the universal screening group was observed to have a significantly longer length of hospitalization, this was driven by several subjects that required inpatient rehabilitation following trauma, prolonged psychiatric counseling, or developed complications related to inflammatory bowel disease. When MDSC comparisons were made in the screening group between those admitted to the ICU and those to the ward, there was a similar trend for higher percentages of T-MDSCs and G-MDSCs in the ICU group, but the differences were not significant, likely related to being underpowered. However, our results indicate that MDSC, specifically G-MDSC, are significantly increased in children with critical illness. This is in agreement with previous reports demonstrating that G-MDSCs are prevalent in acute illnesses while M-MDSCs are prevalent in chronic inflammatory conditions such as cancer or chronic infections (31–35).

Intriguingly, our data also showed that increased circulating MDSCs in children with acute COVID-19 were associated with decreased percentage of CD4 + T cells but increased percentages of CD8 + T cells and NK cells. Both CD4 + and CD8 + T cells also showed higher proportions of activated cells indicated by increased cell surface expression of CD38 and HLA-DR. There was not a significant difference in T regulatory cells between children with < 3% or > 3% MDSC. These findings suggest that in children with COVID-19, MDSCs appear to have a more specific effect on CD4 + T cells, which are crucial for overall orchestration of antiviral immunity and viral clearance (36). MDSCs are known to suppress T cells and inhibit NK cells through direct cell-cell contact and production of chemokines, but those interactions occur at the tissue level (12, 27). Interactions between MDSCs and their target immune cells are complex and dependent on the microenvironment (7, 37, 38). Thus, it is possible that quantifying T cell subsets and NK cells in circulating PBMC does not provide a complete assessment of the overall effect of MDSCs, as it will be important to also assess the presence and function of this cell population in tissue, particularly in the lungs of patients with acute COVID-19. Indeed, while the majority of studies have focused on MDSCs circulating in peripheral blood in these patients, G-MDSCs have also been identified in the lungs of adults who died of COVID-19-related complications (39).

Although confirmatory studies are needed, our preliminary data showed that early circulating MDSCs in children with symptomatic COVID-19 are associated with the need for respiratory support. Of all children hospitalized with acute COVID-19, increased MDSCs were also associated with a significantly longer duration of hospitalization. These data suggest that MDSCs appear to contribute to the pathogenesis and clinical outcomes of severe COVID-19 in children.

Our study has several limitations. First, patients included represent a convenience sample which might have introduced enrollment bias. Nevertheless, patients were enrolled prospectively, and the same approach was used for children hospitalized in the ward and those requiring ICU care, making comparisons fair. Second, we characterized MDSCs by their cell surface markers and we were not able to perform functional studies due to limited blood volume available from children. Third, steroids and other immunomodulatory medications may effect MDSC expansion and function (40). Six of the eight children in the ICU that received COVID-19- directed steroid therapy received this treatment prior to initial blood sample, and all three children admitted to the ward that received steroid treatment had the initial blood sample after treatment as well. Glucocorticoids have been shown to induce MDSC expansion in murine models and may contribute to MDSC expansion observed in our study. Previous studies have indicated that steroid administration is associated with worse outcomes in immunosuppressed phenotypes (41), and it may be that MDSCs play an important role in determining clinical outcomes related to steroid treatment.

A fourth limitation was that our study was not powered to compare differences in immune responses of children who have and have not received vaccinations against COVID-19. During the period of sample enrollment, several SARS-CoV-2 variants, notably delta and omicron- emerged, and effective vaccination programs became available for adults and later children (42). Although previously published data suggest that vaccinations are more effective when adjuvant MSDC-depleting therapies are used (43), it remains unknown what effect the current COVID-19 vaccines have on inflammation-driven MDSC expansion.

Future studies are required to further understand the relationships between MDSCs and target immune cell subsets, such as T cells and NK cells, at the tissue level in the lungs. Earlier and more frequent sampling is needed to explore MDSC dynamics and associations with clinical outcomes in children with COVID-19. MDSCs may have long term effects, including contributing to “long haul COVID,” as this cell population has been found to persist even 3 months after SARS-CoV-2 infection (44). The current methods for identifying MDSC, including PBMC isolation and flow cytometry analysis, are cumbersome, additional studies are needed to identify a reliable serum marker indicating MDSC expansion in children that may be used to stratify patients according to severity of illness. Identifying MDSCs as a potential therapeutic target could allow the development of more personalized treatments plans with already available therapies targeting MDSCs (45). The notably high expression of PD-L1 on G-MDSC and significant increase in this MDSC subset in critically ill children in this study suggests that anti-PDL1 therapy may be a viable option for MDSC inhibition.

## Conclusion

In conclusion, these results indicate that increased MDSCs are part of the immune response of children to SARS-CoV-2 infection. Children with severe COVID-19 had greatly increased total and G-MDSC proportions that were associated with decreased percentage of CD4 + T cells, prolonged hospitalization, and the need for respiratory support, suggesting that this cell population contributes to the pathogenesis of SARS-CoV-2 infection.

## Data Availability Statement

The raw data supporting the conclusions of this article will be made available by the authors, without undue reservation.

## Ethics Statement

The studies involving human participants were reviewed and approved by the Nationwide Children’s Hospital Institutional Review Board. Written informed consent to participate in this study was provided by the participants’ legal guardian/next of kin.

## Author Contributions

KB designed, performed experiments, and wrote the manuscript. AA, SM, and FY performed experiments. VB and ZM assisted with flow cytometry panel design. RS, CT-S, and AQ collected clinical data. MM-C and RG assisted with data analysis. AM and OR reviewed and revised the manuscript. All authors contributed to the article and approved the submitted version.

## Conflict of Interest

The authors declare that the research was conducted in the absence of any commercial or financial relationships that could be construed as a potential conflict of interest.

## Publisher’s Note

All claims expressed in this article are solely those of the authors and do not necessarily represent those of their affiliated organizations, or those of the publisher, the editors and the reviewers. Any product that may be evaluated in this article, or claim that may be made by its manufacturer, is not guaranteed or endorsed by the publisher.

## Supplementary Material

Supplementary Figure 1 Gating strategy for Myeloid Derived Suppressor Cells (MDSCs). MDSCs were identified in fresh PBMC by flow cytometry. After gating on live cells and excluding doublets by FSC-A vs. FSC-H plot, leukocytes were gated by CD45 + vs. SSC-A. MSDCs were then identified by HLA-DR low/− and CD33b+/CD11b+. Subsets were then identified by CD15+/CD14− (G-MDSCs) and CD14+/CD15− (M-MDSCs). Green boxes indicate positive selection.

Supplementary Figure 2 Gating strategy for T cell subsets and NK cells. After gating on lives cells and excluding doublets by FSC-A vs. FSC-H plot, leukocytes were gated by CD45 + vs. SSC-A. T cells were identified by gating on CD3 + cells and then subsets were identified by CD4+/CD8− and CD8−/CD4+. T regulatory cells were CD4 + CD25 + CD127 low. NK cells were identified as CD3-/CD56+. Green boxes indicate positive selection.

Supplementary Figure 3 Example of PD-L1 expression on MDSC subsets. Expression of PD-L1 on both granulocytic-MDSC and monocytic-MDSC subsets identified by PD-L1 vs. SSC-A. Green boxes indicate positive selection.
