# Supplementary figures and images for: Myeloid-Derived Suppressor Cells and Clinical Outcomes in Children With COVID-19
Source: Front Pediatr. 2022 Jun 6;10:893045. doi: 10.3389/fped.2022.893045 (PMC9207271; doi:10.3389/fped.2022.893045)

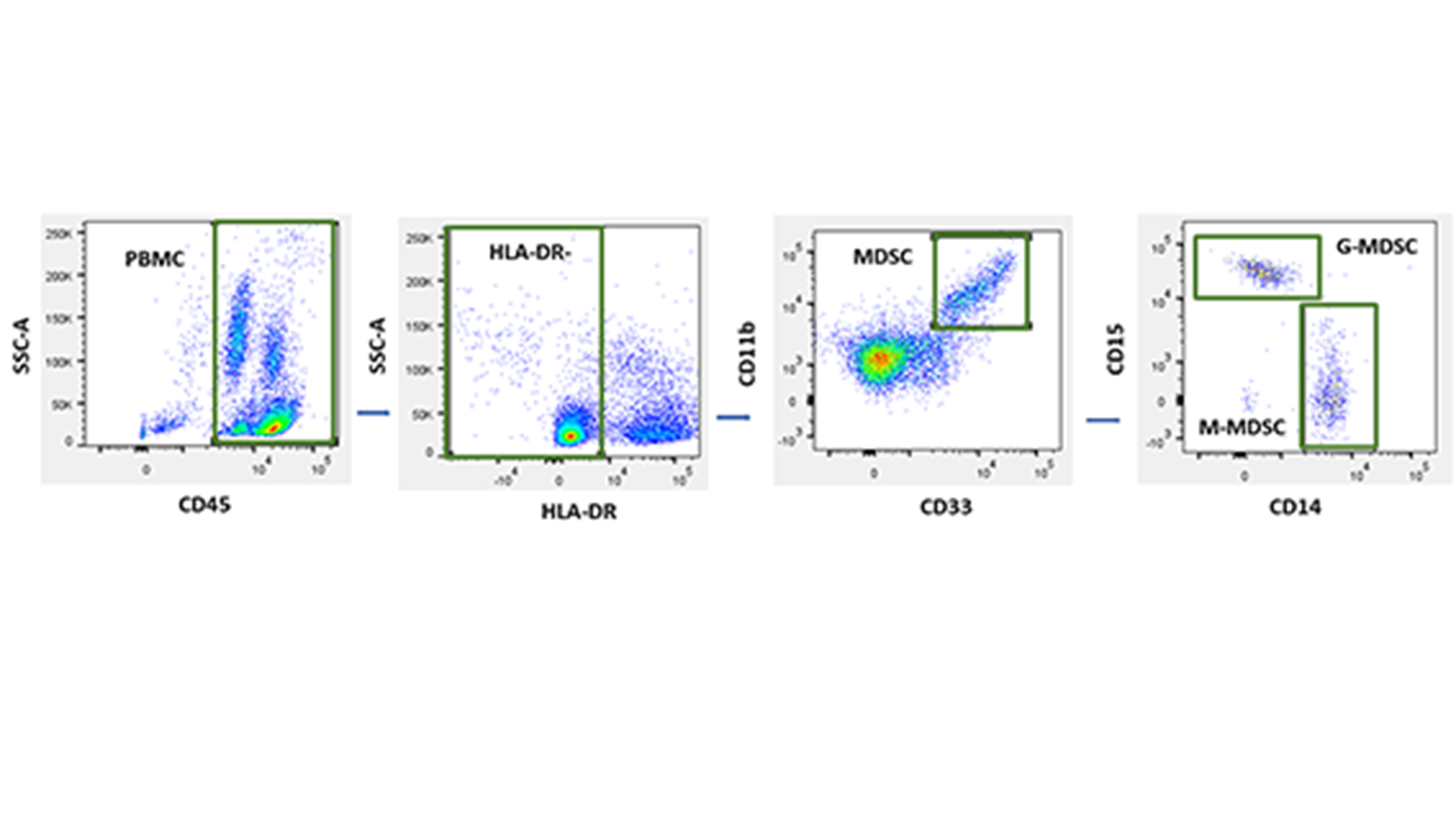

Supplement: Supplementary Figure 1 — Gating strategy for Myeloid Derived Suppressor Cells (MDSCs). MDSCs were identified in fresh PBMC by flow cytometry. After gating on live cells and excluding doublets by FSC-A vs. FSC-H plot, leukocytes were gated by CD45 + vs. SSC-A. MSDCs were then identified by HLA-DR low/− and CD33b+/CD11b+. Subsets were then identified by CD15+/CD14− (G-MDSCs) and CD14+/CD15− (M-MDSCs). Green boxes indicate positive selection. [file Image_1.TIF]

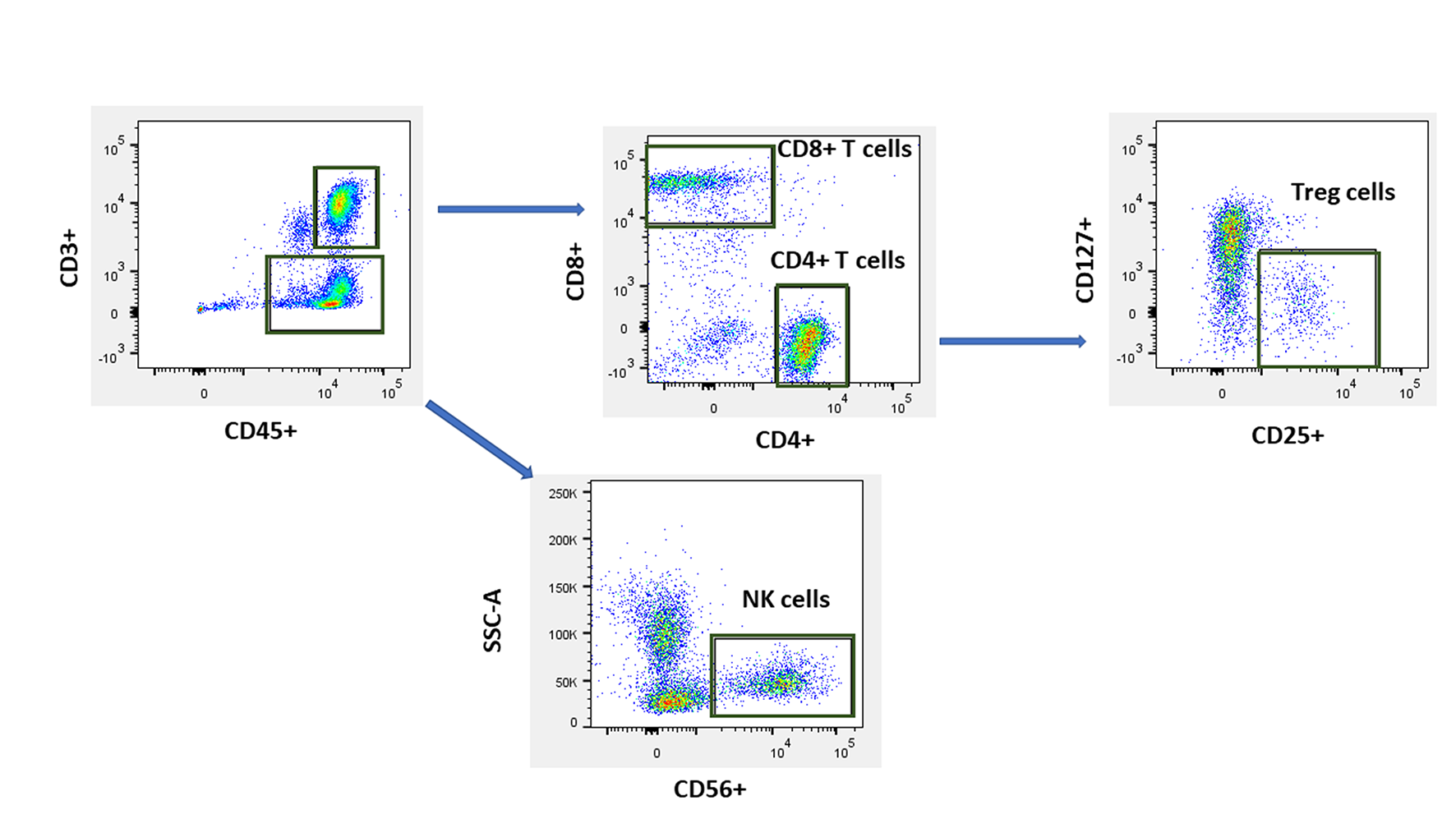

Supplement: Supplementary Figure 2 — Gating strategy for T cell subsets and NK cells. After gating on lives cells and excluding doublets by FSC-A vs. FSC-H plot, leukocytes were gated by CD45 + vs. SSC-A. T cells were identified by gating on CD3 + cells and then subsets were identified by CD4+/CD8− and CD8−/CD4+. T regulatory cells were CD4 + CD25 + CD127 low. NK cells were identified as CD3-/CD56+. Green boxes indicate positive selection. [file Image_2.TIF]

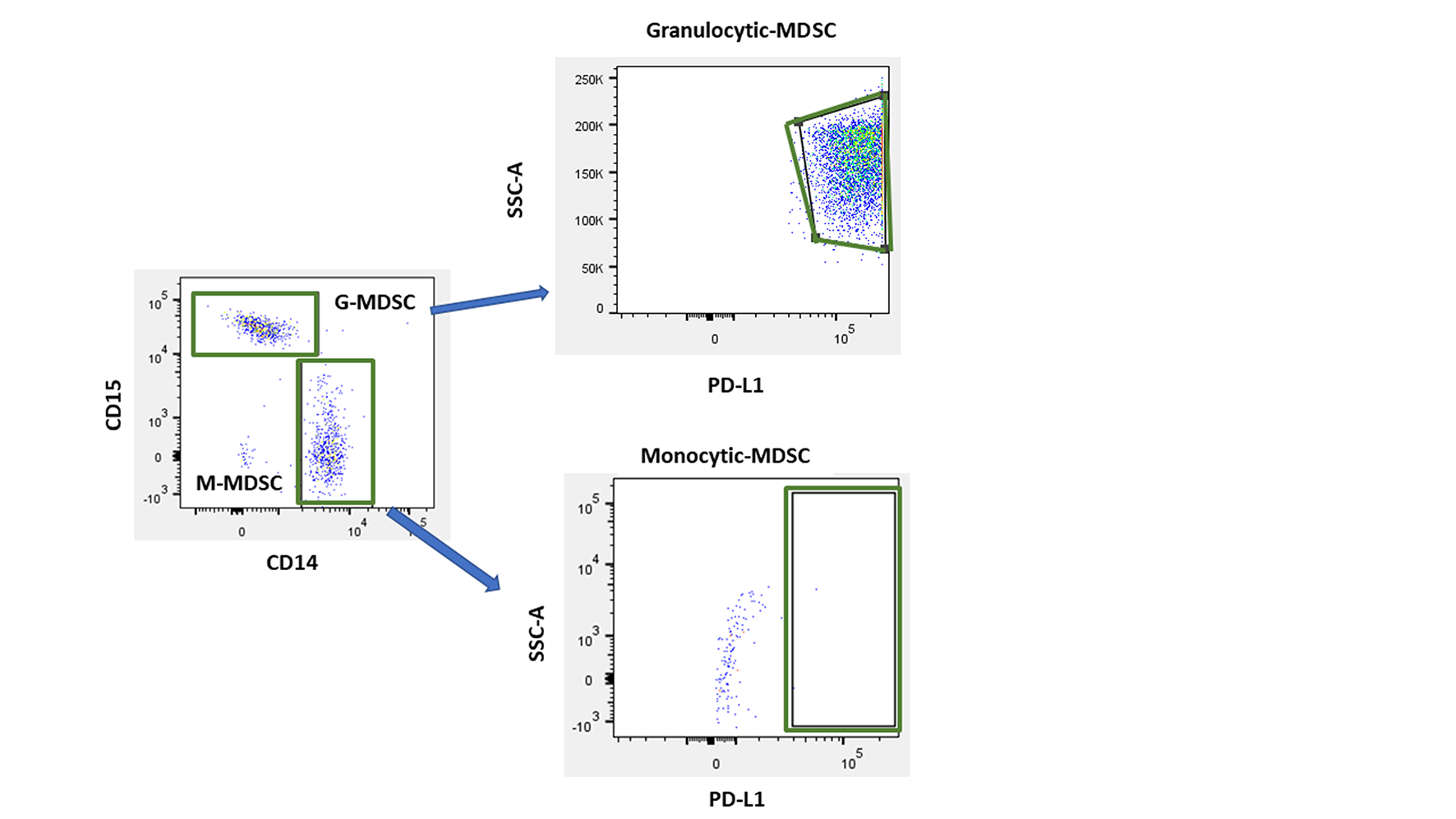

Supplement: Supplementary Figure 3 — Example of PD-L1 expression on MDSC subsets. Expression of PD-L1 on both granulocytic-MDSC and monocytic-MDSC subsets identified by PD-L1 vs. SSC-A. Green boxes indicate positive selection. [file Image_3.tif]
